# Overall Postoperative Morbidity and Pancreatic Fistula Are Relatively Higher after Central Pancreatectomy than Distal Pancreatic Resection: A Systematic Review and Meta-Analysis

**DOI:** 10.1155/2020/7038907

**Published:** 2020-02-22

**Authors:** Parbatraj Regmi, Qing Yang, Hai-Jie Hu, Fei Liu, Hare Ram Karn, Wen-Jie Ma, Cong-Dun Ran, Fu-Yu Li

**Affiliations:** ^1^Department of Biliary Surgery, West China Hospital of Sichuan University, Chengdu 610041, Sichuan Province, China; ^2^Department of General Surgery, The Third People's Hospital of Chengdu, The Affiliated Hospital of Southwest Jiaotong University, The Second Medical School of Chengdu Affiliated to Chongqing Medical University, Chengdu, Sichuan 610031, China

## Abstract

**Objective:**

To compare the intraoperative and postoperative outcomes of central pancreatectomy (CP) with distal pancreatectomy (DP).

**Methods:**

A systematic literature search was performed on electronic databases from MEDLINE, Embase, and PubMed from 1998 to 2018. Statistical analysis and meta-analysis were performed using statistics/data analysis (Stata®) software, version 12.0 (StataCorp LP, College Station, Texas 77845, USA). Dichotomous variables were analyzed by estimation of relative risk (RR) with a 95 percent (%) confidence interval (CI) and continuous variables were analyzed by standardized mean differences (SMD) with 95% CI.

**Results:**

Twenty-four studies with 593 CP and 1226 DP were included in the meta-analysis. CP had significantly longer operation time (SMD: 1.03; 95% CI 0.62 to 1.44; *P* < 0.001) and lengthier postoperative hospital stay (SMD: 0.63; 95% CI 0.20 to 1.05; *P* < 0.001) and lengthier postoperative hospital stay (SMD: 0.63; 95% CI 0.20 to 1.05; *P* < 0.001) and lengthier postoperative hospital stay (SMD: 0.63; 95% CI 0.20 to 1.05; *P* < 0.001) and lengthier postoperative hospital stay (SMD: 0.63; 95% CI 0.20 to 1.05; *P* < 0.001) and lengthier postoperative hospital stay (SMD: 0.63; 95% CI 0.20 to 1.05; *P* < 0.001) and lengthier postoperative hospital stay (SMD: 0.63; 95% CI 0.20 to 1.05; *P* < 0.001) and lengthier postoperative hospital stay (SMD: 0.63; 95% CI 0.20 to 1.05; *P* < 0.001) and lengthier postoperative hospital stay (SMD: 0.63; 95% CI 0.20 to 1.05; *P* < 0.001) and lengthier postoperative hospital stay (SMD: 0.63; 95% CI 0.20 to 1.05; *P* < 0.01). Estimated blood loss was significantly lower in CP (SMD: −0.34; 95% CI −0.58 to −0.09; *P* = 0.007). Overall postoperative morbidity (RR: 1.30; 95% CI: 1.13 to 1.50; *P* < 0.001), overall pancreatic fistula (RR: 1.41; 95% CI: 1.20 to 1.66; *P* < 0.001), clinically relevant fistula (RR: 1.64; 95% CI: 1.25 to 2.16; *P* < 0.001), and postoperative hemorrhage (RR: 1.90; 95% CI: 1.18 to 3.06; *P* < 0.05) were all significantly higher after CP. On long-term follow-up, DP patients were more likely to have postoperative exocrine (RR: 0.56; 95% CI: 0.37 to 0.84; *P* < 0.05) and endocrine (RR: 0.27; 95% CI: 0.18 to 0.40; *P* < 0.001) insufficiency. There was no statistically significant difference in transfusion requirement, postoperative mortality, reoperation, and tumor recurrence.

**Conclusion:**

CP is associated with significantly higher morbidity and clinically relevant pancreatic fistula. CP should only be reserved for selected patients who require postoperative pancreatic function preservation.

## 1. Introduction

Complete surgical resection is the only potentially curative treatment for pancreatic cancer. However, only 15–20% of patients are amenable to resection on initial diagnosis [1–3]. The distal pancreatectomy (DP) is considered as a standard surgical procedure for lesions located in the pancreatic neck and body [2]. Unfortunately, during the resection of benign and low-malignant lesions, normal pancreatic parenchyma is resected in the DP and may result in loss of pancreatic function and possible postoperative exocrine and endocrine impairment. After the introduction of the first central pancreatectomy (CP) with reconstruction by Dagradi and Serio in 1982, this procedure has been used as a parenchyma-preserving surgical procedure for resection of benign and low-malignant lesions of neck and proximal body of pancreas. After that, the procedure has been advanced gradually from open surgery to laparoscopic and robotic approaches [4, 5].

Current literature has reported a relatively higher incidence of postoperative new-onset diabetes mellitus after DP than CP and pancreaticoduodenectomy (PD) [6, 7]. Kang et al. have reported that resected pancreatic volume was an independent risk factor for postoperative endocrine impairment [8]. In CP, the volume of remnant pancreas is responsible for maintaining postoperative endocrine and exocrine function, but there are still controversies regarding the management of additional pancreatic stump. The morbidity following CP is comparatively higher than other standard pancreatic resections, pancreatic fistula being major morbidity [9, 10]. CP has relative benefits of preserving normal pancreatic parenchyma and spleen, but potential challenges to reconstruct additional pancreatic stump and high incidence of postoperative pancreatic fistula create a dilemma to choose appropriate surgical procedure.

Expected benefits and potential complications create controversies in selecting surgical procedures for lesions of the pancreatic neck and proximal body, so this study was aimed to review all the relevant electronic databases to evaluate and compare intraoperative, short- and long-term, and postoperative outcomes following CP and DP.

## 2. Methods

The systematic review and meta-analysis were performed according to the Preferred Reporting Items for the Systematic Review and Meta-Analysis Protocols (PRISMA-P) guideline [11].

### 2.1. Search Strategy

**Figure 1:**
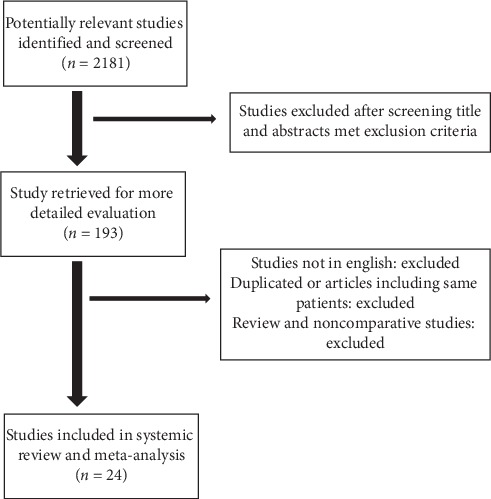
Prisma flow chart.

A systematic literature search was performed on electronic databases from Ovid MEDLINE (R), Embase, and PubMed from 1 January 1998 to 31 December 2017. Search headings used were “(central pancreatectomy OR medial pancreatectomy OR middle pancreatectomy OR segmental pancreatectomy) and (distal pancreatectomy OR left pancreatectomy)”. Searches were performed without any restrictions and all the abstracts, studies, and citations were reviewed. 2181 studies were found after the comprehensive search of the database. 1988 articles were excluded after screening the title abstracts and duplicated materials. 193 studies were evaluated in detail and ultimately 24 eligible studies were included in systematic review and meta-analysis (Figure 1).

### 2.2. Study Selection and Quality Assessment

Two reviewers independently screened all the selected citations independently. Any disagreement between the two reviewers was resolved by discussion with the corresponding author. All the retrospective and prospective matched pairs and nonmatched pairs comparing CP and DP were extracted.

Inclusion criteria were

1. Original English articles;
2. Patients with benign pathology of low-malignant tumors of the pancreatic neck or proximal body;
3. Studies comparing the clinical outcomes between CP and DP;
4. Studies that provided adequate information about demographic characteristics and intraoperative and postoperative outcomes.

Exclusion criteria were

1. Original articles with <5 CP or DP patients;
2. Abstracts, expert opinion, reviews, editorials, and letter to the editor;
3. Studies that lack adequate clinical data on intraoperative and postoperative outcomes.

Quality assessment was done by the Newcastle Ottawa Scale (NOS). Studies selected had a score above 5. Scoring criteria were based on the selection of study groups, the comparability of groups, and the ascertainment of either exposure or outcome. One study had 9, 3 had 8, 17 had 7, and 3 had 6 out of possible 9 scores.

### 2.3. Data Extraction

Data were extracted for (a) demographic characteristics, (b) intraoperative outcomes (operation time, intraoperative blood loss, and transfusion requirement), (b) short-term postoperative outcomes (postoperative hospital stay, overall morbidity, pancreatic fistula, clinically relevant pancreatic fistula, postoperative hemorrhage, reoperation, and 30-day mortality), and (c) long-term outcomes (overall endocrine function, Insulin-dependent diabetes mellitus, exocrine function, and tumor recurrence). If two articles were published by the same authors or from the same institution, a comparatively more informative study with the maximum population was selected. When data was found in median and range, the mean and standard deviation was estimated as described by Wan et al. and Luo et al. [12, 13].

### 2.4. Statistical Analysis

Statistical analysis and meta-analysis were performed by statistics/data analysis (Stata®) software, version 12.0 (StataCorp LP, College Station, Texas 77845 USA). Dichotomous variables were analyzed by estimation of relative risk (RR) with a 95 percent (%) confidence interval (CI) and continuous variables were analyzed by standardized mean differences (SMD)/weighted mean differences (WMD) with 95% CI. *P* value <0.05 was considered a statistically significant difference between the two groups. Heterogeneity was defined as low, moderate, and high based on *I* square value (<25%: low; 25–75%: moderate; >75%: high). Heterogeneity with a high *I* square value >30% and *P* value <0.05 was considered statistically significant. Fixed effect (Mantel–Haenszel) model was used when there was no significant heterogeneity and the random effects (DerSimonian and Laird) model was used for those with significant heterogeneity. Publication bias was examined in a funnel plot using Begg's and Egger's tests. Publication bias was considered to be present when the *P* value was <0.1.

## 3. Results

### 3.1. Study Selection and Characteristics

Twenty-four studies performed from 1998 to 2018 were included in meta-analysis following a comprehensive literature search (Table 1). Central pancreatectomy and distal pancreatectomy were performed in 593 and 1226 patients, respectively. Of these 24 studies, five studies were performed in Japan [14–18], three each in China [19–21], USA [7, 22, 23], and Korea [24–26], two each in Germany [27, 28] and Italy [29, 30], one each in Taiwan [31], Spain [32], Netherlands [33], Romania [34], and France [35], and one combined study in USA and Italy [36]. Among them, two studies were prospective [28, 32] and others were retrospective. Five studies were performed on minimally invasive procedures (three robotic [21, 23, 30] and two laparoscopic [26, 35]). Indications for the central or distal pancreatectomy were benign pathologies like trauma and pancreatitis, benign and borderline tumors of pancreatic neck and proximal body, and few malignant cases (Table 2).

## 4. Quantitative Data Synthesis

Results of meta-analysis are included in Table 3.

### 4.1. Intraoperative Outcomes

**Figure 2:**
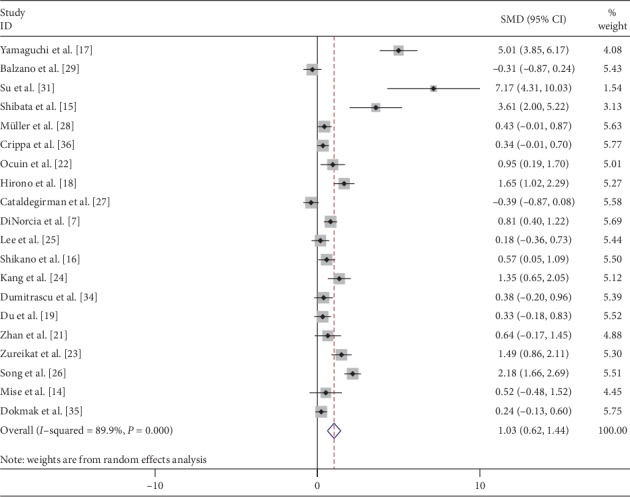
Forest plot comparing duration of operation for central versus distal pancreatectomy.

**Figure 3:**
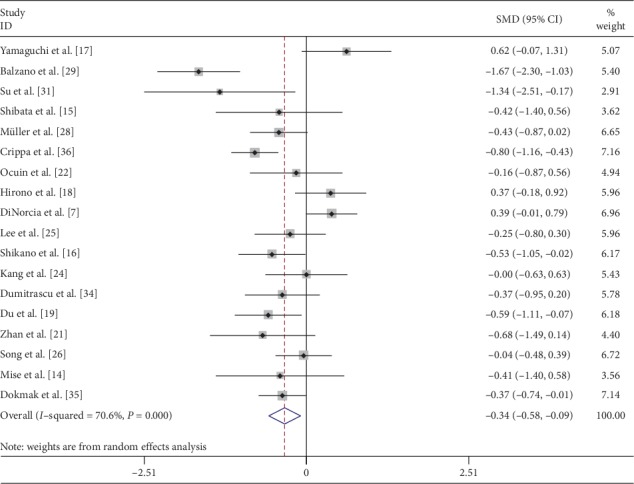
Forest plot comparing estimated blood loss for central versus distal pancreatectomy.

**Figure 4:**
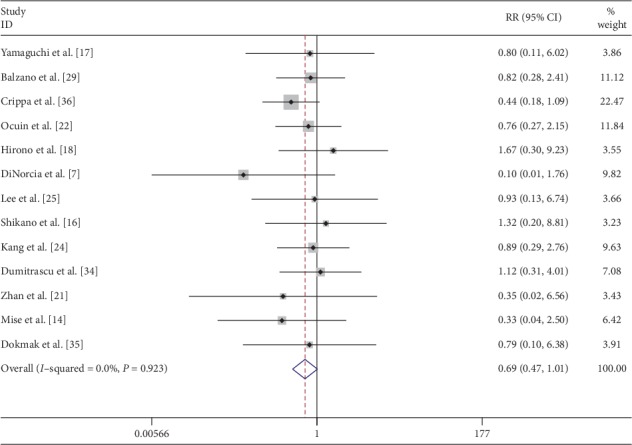
Forest plot comparing transfusion requirement for central versus distal pancreatectomy.

Operation time was pooled from 20 [7, 14–19, 21–29, 31, 34–36] studies and meta-analysis was done with the random effects model. CP was associated with significantly longer operation time (SMD: 1.03; 95% CI 0.62 to 1.44; *P* < 0.001) (Figure 2). CP was associated with significantly less blood loss when data were pooled from 18 [7, 14–19, 21, 22, 24–26, 28, 29, 31, 34–36] comparative studies with random effects model (SMD: −0.34; 95% CI −0.58 to −0.09; *P*=0.007) (Figure 3). Transfusion requirement was pooled from 13 [7, 14, 16–18, 21, 22, 24, 25, 29, 34–36] comparative studies, but the difference was not statistically significant (SMD: 0.69; 95% CI 0.47 to 1.01; *P*=0.059) (Figure 4).

### 4.2. Postoperative Outcomes

**Figure 5:**
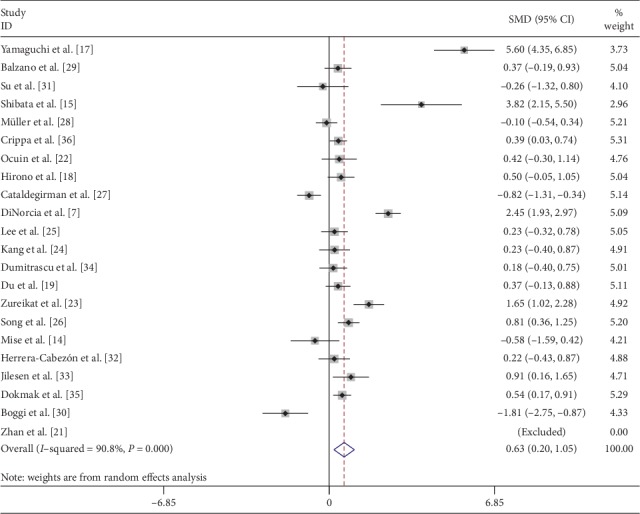
Forest plot comparing length of hospital stay (LOS) for central versus distal pancreatectomy.

Length of postoperative hospital stay (LOS) was compared in 23 [7, 14, 15, 17–34, 36, 37] studies and meta-analysis was done using a random effects model. CP had significantly longer postoperative LOS (SMD: 0.63; 95% CI 0.20 to 1.05; *P* < 0.01) (Figure 5).

**Figure 6:**
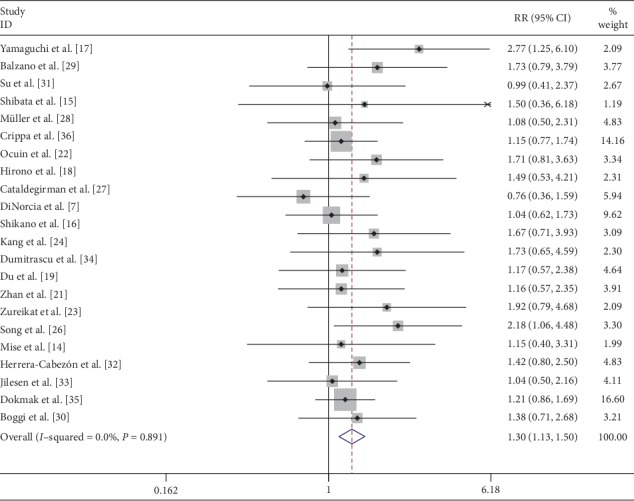
Forest plot comparing overall postoperative morbidity for central versus distal pancreatectomy.

**Figure 7:**
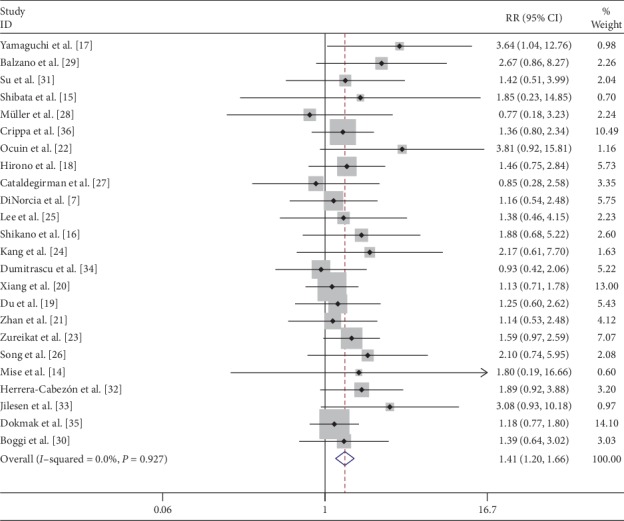
Forest plot comparing overall pancreatic fistula for central versus distal pancreatectomy.

Overall postoperative morbidity was recorded in 50.3% (251/499) of patients following CP and 39.2% (393/1002) of patients following DP. The most common complication was a postoperative pancreatic fistula. Reported data on overall complications in 21 [7, 14–18, 21–24, 26–36] studies showed that the complications were significantly higher after CP (RR: 1.30; 95% CI: 1.13 to 1.50; *P* < 0.001) than those after DP (Figure 6). Similarly, the overall pancreatic fistula was significantly higher after CP (RR: 1.41; 95% CI: 1.20 to 1.66; *P* < 0.001) [7, 14–36] (Figure 7). Overall pancreatic fistulas following CP and DP were 39.6% (235/593) and 26.3% (323/1226), respectively. Classification of the fistula was done according to the International Study Group for Pancreatic Fistula (ISGPF), Clavien–Dindo classification, and amount of postoperative amylase drainage in some studies. Most of the pancreatic fistulas following CP or DP were successfully treated by percutaneous drainage and antibiotics.

**Figure 8:**
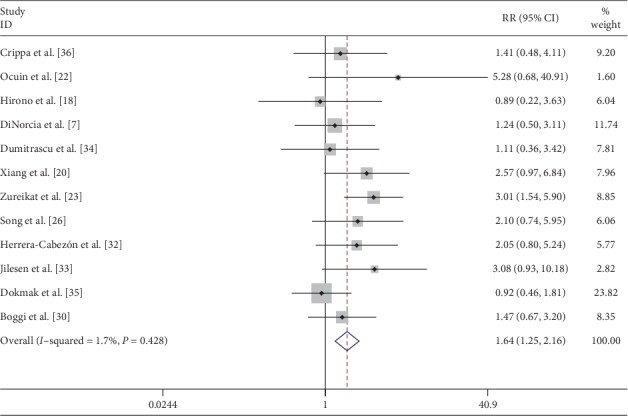
Forest plot comparing clinically relevant pancreatic fistula (Grade B + C) for central versus distal pancreatectomy.

**Figure 9:**
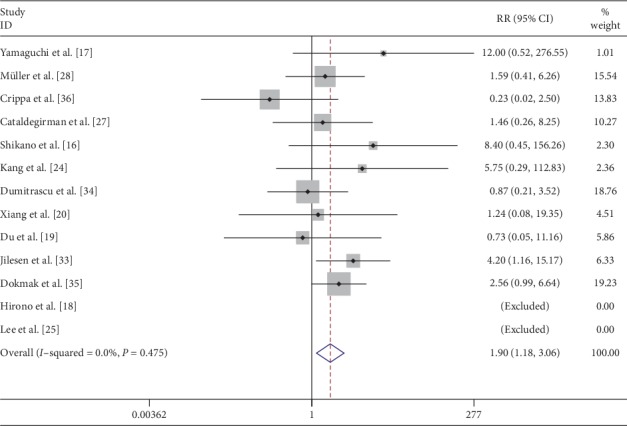
Forest plot comparing postoperative hemorrhage (POH) for central versus distal pancreatectomy.

Classification of the fistula was recorded in 12 [7, 18, 20, 22, 23, 26, 30, 32–36] studies with 345 and 825 patients undergoing CP and DP, respectively. Grade A fistulas following CP and DP were seen in 65 (18.8%) and 81 (9.8%) patients, respectively. Clinically relevant fistulas (Grade B + C) were seen in 23.1% (81/350) and 18.18% (150/825) patients with CP and DP, respectively. Pooled data from these studies showed that there was a significantly higher incidence of clinically relevant fistula after CP (RR: 1.64; 95% CI: 1.25 to 2.16; *P* < 0.001) (Figure 8). The data pooled from 11 studies indicated that CP had significantly higher risk of postoperative hemorrhage (RR: 1.90; 95% CI: 1.18 to 3.06; *P* < 0.05) (Figure 9).

**Figure 10:**
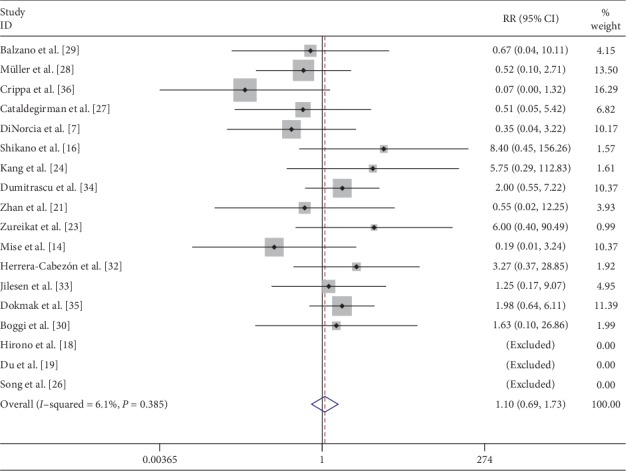
Forest plot comparing reoperation for central versus distal pancreatectomy.

Reoperation was compared in 15 [7, 14, 16, 21, 23, 24, 27–30, 32–36] studies, which showed that there was no significant difference in risk of reoperation between two groups (RR: 1.10; 95% CI: 0.69 to 1.73) (Figure 10). Reoperation was performed in 23 patients who underwent CP and 44 patients who underwent DP. Causes of reoperation after CP include postoperative hemorrhage (*n* = 10), anastomotic insufficiency (*n* = 2), splenic infarction (*n* = 1), tracheostomy for prolonged ventilation (*n* = 1), and unknown (*n* = 9). Causes of reoperation in DP were postoperative hemorrhage (*n* = 9), anastomotic insufficiency (*n* = 3), postoperative bowel obstruction (*n* = 2), intra-abdominal abscess (*n* = 2), splenic infarction (*n* = 1), ischemic cecal perforation (*n* = 1), wound dehiscence (*n* = 1), and unknown (*n* = 25). In unknown causes of reoperation, causes were not mentioned clearly. Mortality data were compared in 18 studies. Two cases of 30-day mortality were seen after CP, one with postoperative hemorrhage and the other secondary to pulmonary failure. Only one case of 30-day mortality secondary to myocardial infarction was reported after DP. Comparative data of tumor recurrence was mentioned in 10 studies. Nine cases of recurrence were identified after CP and 21 cases after DP.

Data on perioperative mortality (30 days postoperative) was extracted from 3 [22, 28, 32] studies. There were two mortalities in the CP group and one in the DP group. The cause of death after CP was postoperative hemorrhage and pulmonary failure. The patient in the DP group died due to myocardial infarction. Pooled data showed that mortality in the two groups had no significant difference (RR: 3.31; 95% CI: 0.52 to 21.32; *P*=0.207) (Supplementary [Supplementary-material supplementary-material-1]).

### 4.3. Long-Term Outcomes

**Figure 11:**
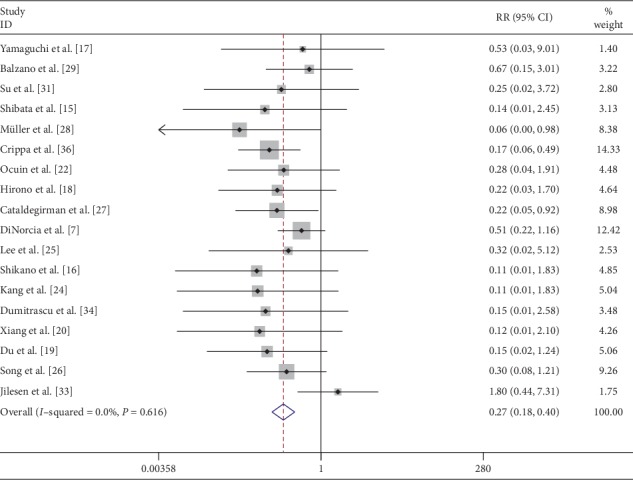
Forest plot comparing postoperative endocrine insufficiency for central versus distal pancreatectomy.

The postoperative endocrine function was assessed with the assessment of antidiabetic treatment, fasting blood glucose, hemoglobin A1c levels, oral glucose tolerance test, and World Health Organization criteria. All patients with preoperative diabetes were removed from the analysis. Overall incidence of postoperative new-onset diabetes was recorded in 18 [7, 15, 16, 18–20, 22–29, 31, 33, 34, 36] studies and was noted in 4.8% (23/483) CP patients and 22.05% (153/694) DP patients. The pooled data of these studies showed that the risk of postoperative endocrine function impairment was significantly lower after CP (RR: 0.27; 95% CI: 0.18 to 0.40; *P* < 0.001) (Figure 11). Data of insulin-dependent diabetes mellitus (IDDM) was compared in 6 [7, 16, 18, 19, 22, 36] studies and the pooled data demonstrated that patients in CP group were less likely to suffer from IDDM (RR: 0.15; 95% CI: 0.06 to 0.42; *P* < 0.001) (Supplementary [Supplementary-material supplementary-material-1]).

**Figure 12:**
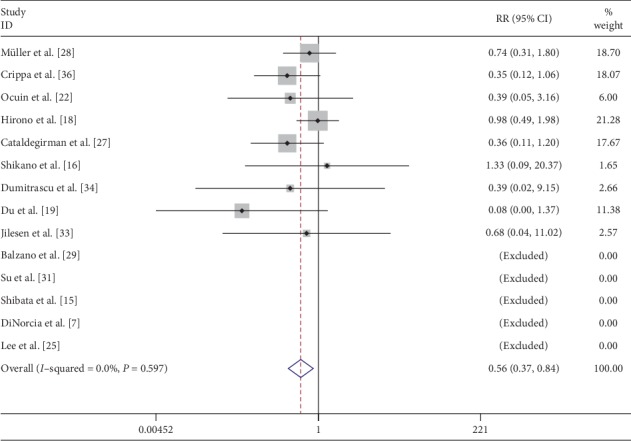
Forest plot comparing postoperative exocrine failure for central versus distal pancreatectomy.

The postoperative exocrine function was assessed by laboratory tests (p-aminobenzoic test, fecal chymotrypsin level, and pancreolauryl test), symptoms (weight loss, diarrhea, and steatorrhoea), and the need for pancreatic enzyme supplementation. Postoperative exocrine insufficiency was recorded in 9 [16, 18, 19, 22, 27, 28, 33, 34, 36] studies with 8.9% (27/304) and 17.3% (56/324) following CP and DP, respectively. Pooled data of these studies indicated that patients in the CP group were less likely to suffer from exocrine insufficiency (RR: 0.56; 95% CI: 0.37 to 0.84; *P* < 0.05) (Figure 12).

Comparative data of tumor recurrence was pooled from 7 [16, 21, 25, 27, 28, 33, 34] studies suggesting no significant difference between the two groups (RR: 1.02; 95% CI: 0.48 to 2.20; *P* > 0.05) (Supplementary [Supplementary-material supplementary-material-1]).

## 5. Heterogeneity and Publication Bias

Significant heterogeneity (high *I* square value >30% and *P* value <0.05) was observed in three continuous variables (operation time, estimated blood loss, and length of hospital stay). All three outcomes were pooled on the random-effects model. Therefore, a sensitivity analysis was done by omitting 1 study at a time and the pooled RR was calculated for the remaining studies to identify the potential source of heterogeneity between studies, but no single study significantly affected the primary outcome or heterogeneity. This may be due to a difference in surgical skills among surgeons and postoperative management strategy. Publication bias was considered to be present when the *P* value was <0.1. Assessment of publication bias of dichotomous data using the funnel plot showed symmetrical distribution and the Egger's test did not show any statistical significance.

## 6. Discussion

This meta-analysis included 24 studies involving 1819 patients and assessed the intraoperative and postoperative outcomes after CP or DP. Long operation time in the CP is due to its distinct anatomical location and complex surgical procedure. But one [29] study reported a comparatively shorter duration of operation in the CP group. In our study, on comparing intraoperative outcomes between the two procedures, estimated blood loss was statistically higher during distal pancreatectomy. Thus, a relatively higher number of patients required transfusion after distal pancreatectomy. In CP, the method of reconstruction of the distal stump was recorded in 17 studies with 359 patients. Pancreaticojejunostomy (PJ) was performed in 225 patients and pancreaticogastrostomy in 112 patients. The remaining 22 cases were treated with duct occlusion. In DP, the proximal stump was ligated in most of the cases, but, in a few cases, anastomosis of a remnant of the pancreatic head with the Roux-en-Y limb was done. The rate of fistula following different reconstruction techniques was not compared in most of the studies.

The difference in the cumulative incidence of overall postoperative morbidity after CP and DP was 50.3% and 39.2%, respectively. Huge variation was observed in morbidity after CP among included studies ranging from 25.7% [27] to 92%. The overall incidence of the postoperative pancreatic fistula and the clinically relevant postoperative pancreatic fistula was significantly higher after CP. In CP there is the formation of two stumps, reconstruction with two ductal-enteric anastomoses, and therefore a high risk of a pancreatic leak from the anastomosis. A recent meta-analysis by Ricci et al. indicated that the risk of the clinically relevant pancreatic fistula is relatively higher after reconstruction with PJ [38]. This may be a cause of a high incidence of postoperative pancreatic fistula after CP as PJ was performed in the majority of patients in our study. Clinically relevant fistula did not differ significantly between two groups in the previous meta-analysis (RR: 0.76; 95% CI: 0.37–1.57; *P* > 0.05) [39], but our study showed significantly high incidence of clinically relevant pancreatic fistula (Grade B + C) (RR: 1.64; 95% CI: 1.25 to 2.16; *P* < 0.001). For patient's safety, most of the intraoperative and postoperative outcomes favor DP over CP.

The incidence of postoperative hemorrhage was significantly high in the CP group. We believe segmental resection of a relatively large pancreatic neck tumor exposes an area of the portal vein (PV) near ducto-enteric anastomosis, hence, a pancreatic leak that may erode surrounding blood vessels and induce postoperative hemorrhage. In our study, 10 out of 23 CP patients required reoperation for postoperative hemorrhage, but only 9 out of 44 DP patients required reoperation for postoperative hemorrhage. Complex reconstruction techniques and exposed surrounding blood vessels might be the reason for reoperation in a relatively higher percentage of CP patients with PO hemorrhage. However, there was no significant difference in the overall rate of reoperation between the two groups. 30-day postoperative mortality was also higher in the CP group which was not statistically significant.

A significant difference in the incidence of postoperative new-onset endocrine insufficiency was seen between CP and DP (4.8% versus 22.05%). Postoperative endocrine insufficiency was not observed in 9 studies [15, 17, 20, 24, 25, 28, 31, 34] after CP, but it was observed in all studies after DP. A recent nationwide database study on glucose metabolism after DP has shown 22.1% incidence of new-onset diabetes. [40] Female gender, higher BMI, and resection of pancreatic volume >25% are the risk factors for postoperative endocrine impairment [8]. Incidence of postoperative endocrine insufficiency following DP was 19.5% in studies performed in the Asian population [15–20, 24–26, 31] and 26.5% in studies performed in the western population [7, 22, 27–29, 33, 34, 36]. The difference incidence of new-onset IDDM was even greater, for which the cumulative incidence was 0.9% (2/227) after CP and 12.9% (21/163) after DP [7, 16, 18, 19, 22, 36]. The previous meta-analysis showed no significant difference in the incidence of postoperative exocrine failure (pooled RR: 0.59; 95% CI: 0.32 to 1.07; *P*=0.082) [41]. In contrast, our study showed a significant difference between the two groups (RR: 0.56; 95% CI: 0.37 to 0.84; *P* < 0.05). The incidence of postoperative exocrine failure after CP or CP varies widely, as it depends on preexisting pancreatic abnormality, the extent of resection, and presence of chronic pancreatitis [28, 42, 43]. Therefore, in selected patients who need preservation of the pancreatic parenchyma, CP is of utmost importance.

Tumor recurrence was observed in 1.52% of patients following CP, among whom the majority of cases were IPMN. A study has shown that mucinous neoplasms have a 62.2% positive predictive value for malignancy, so intraoperative frozen-section analysis is necessary to obtain R0 resection and reduce the incidence of tumor recurrence [44, 45].

## 7. Conclusion

Our study suggests that pancreatic neck resection requires longer operation time, high rate of postoperative pancreatic fistula, and high morbidity and mortality, but less amount of normal parenchyma is resected in contrast to DP. The incidence of postoperative endocrine and exocrine insufficiency is relatively less after CP. Few previously published meta-analyses have shown that CP can be feasible for benign and low-malignant lesions of the pancreatic neck and proximal body. In contrast to those studies, our study showed that the incidence of serious postoperative morbidity (i.e., clinically relevant pancreatic fistula) was significantly high after CP. In our study, the cumulative incidence of postoperative endocrine insufficiency was relatively lower in Asians compared to the western population (19.5% versus 26%). We believe that postoperative diabetes can be well controlled with oral hypoglycemic drugs and insulin. However, regarding higher morbidity and mortality after CP, it is still questionable for patient safety.

Although CP has the advantage of postoperative pancreatic function preservation, due to lengthy operation time, high rate of complications, and higher incidence of postoperative fistula. We conclude DP is a comparatively safe procedure compared to CP. Therefore, for tumors in pancreatic body and tail, DP is the safest, most feasible, and accepted procedure unless pancreatic parenchyma preservation is of utmost importance.

## Figures and Tables

**Table 1:** Baseline characteristics of included studies.

Authors	Country	Year	Group (CP/DP)	Gender (M/F)	Approach	Study type	Quality scores
Yamaguchi et al. [17]	Japan	2000	10/47	27/30	Open	Retrospective	
Balzano et al. [29]	Italy	2003	32/21	16/37	Open	Retrospective	
Su et al. [31]	Taiwan	2004	5/11	—	Open	Retrospective	
Shibata et al. [15]	Japan	2004	10/7	6/11	Open	Retrospective	
Müller et al. [28]	Germany	2006	40/40	38/42	Open	Prospective	
Crippa et al. [36]	Italy and USA	2007	100/45	37/108	Open	Retrospective	
Ocuin et al. [22]	USA	2008	13/18	8/23	Open	Retrospective	
Hirono et al. [18]	Japan	2009	24/28	19/33	Open	Retrospective	
Cataldegirmen et al. [27]	Germany	2010	35/35	34/36	Open	Retrospective	
DiNorcia et al. [7]	USA	2010	50/50	26/74	Open	Retrospective	
Lee et al. [25]	Korea	2010	14/143	56/101	Open	Retrospective	
Shikano et al. [16]	Japan	2010	26/35	30/31	Open	Retrospective	
Kang et al. [24]	Korea	2011	17/22	15/24	Open	Retrospective	
Dumitrascu et al. [34]	Romania	2012	22/25	11/36	Open	Retrospective	
Xiang et al. [20]	China	2012	44/45	36/63	Open	Retrospective	
Du et al. [19]	China	2013	36/26	20/42	Open	Retrospective	
Zhan et al. [21]	China	2013	10/16	—	Robotic	Retrospective	
Zureikat et al. [23]	USA	2013	13/83	—	Robotic	Retrospective	
Song et al. [26]	Korea	2014	26/96	34/88	Laparoscopic	Retrospective	
Mise et al. [14]	Japan	2014	8/8	12/4	Open	Retrospective	
Herrera-Cabezón et al. [32]	Spain	2015	10/105	57/58	Open	Prospective	
Jilesen et al. [33]	Netherlands	2015	8/72	30/50	Open	Retrospective	
Dokmak et al. [35]	France	2017	35/165	74/126	Laparoscopic	Retrospective	
Boggi et al. [30]	Italy	2016	5/83	—	Robotic	Retrospective	

**Table 2:** Results of meta-analysis: CP versus DP.

Outcome of interest	Studies no.	Patients no. (CP/DP)	RR or SMD	95% CI	*P* value	*I* ^2^ (%)	Model
Intraoperative outcomes							
Operation time (min)	20	526/911	1.03	(0.62, 1.44)	<0.001	90%	Random effects
EBL (ml)	18	478/793	−0.34	(−0.58,−0.09)	0.007	70.6%	Random effects
Transfusion (*n*)	13	361/623	0.69	(0.47, 1.01)	0.059	0.0	Fixed effects
Postoperative outcomes							
LOS (days)	23		0.63	(0.20, 1.05)	<0.01	90.8%	Random effects
Overall complications	21	499/1002	1.30	(1.13, 1.50)	<0.001	0.0	Fixed effects
Overall PF	24	593/1226	1.41	(1.20, 1.66)	<0.001	0.0	Fixed effects
PF (grade B + C)	12	350/825	1.64	(1.25, 2.16)	<0.001	1.7	Fixed effects
POH	11	373/567	1.90	(1.18, 3.06)	0.008	0.0	Fixed effects
Reoperation (*n*)	15	411/805	1.10	(0.69, 1.73)	0.699	6.1	Fixed effects
Perioperative mortality	3	63/163	3.31	(0.52, 21.32)	0.207	0.0	Fixed effects
Long-term outcomes							
Endocrine impairment	18	483/694	0.27	(0.18, 0.40)	<0.001	0.0	Fixed effects
IDDM	6	227/163	0.15	(0.06, 0.42)	<0.001	0.0	Fixed effects
Exocrine impairment	9	304/324	0.56	(0.37, 0.84)	0.006	0.0	Fixed effects
Recurrence	7	155/366	1.02	(0.48, 2.20)	0.956	0.0	Fixed effects

EBL: estimated blood loss; LOS: length of hospital stay; PF: pancreatic fistula; POH: postoperative hemorrhage; IDDM: insulin-dependent diabetes mellitus.

**Table 3:** Surgical pathology of included studies.

Authors	Surgical pathology	
	Benign or borderline (CP/DP)	Malignant (CP/DP)
Yamaguchi et al. [17]	10/47	0/0
Balzano et al. [29]	32/21	0/0
Su et al. [31]	5/11	0/0
Shibata et al. [15]		
Müller et al. [28]	36/36	4/4
Crippa et al. [36]	93/17	7/28
Ocuin et al. [22]	13/18	0/0
Hirono et al. [18]	20/24	4/4
Cataldegirman et al. [27]	32/34	3/1
DiNorcia et al. [7]	50/46	0/4
Lee et al. [25]	14/121	0/22
Shikano et al. [16]	24/33	2/2
Kang et al. [24]	17/22	0/0
Dumitrascu et al. [34]	19/21	3/4
Xiang et al. [20]	44/55	0/0
Du et al. [19]	36/26	0/0
Zhan et al. [21]	10/12	0/4
Zureikat et al. [23]	13/6	0/77
Song et al. [26]	26/96	0/0
Mise et al. [14]	8/8	0/0
Herrera-Cabezón et al. [32]	10/82	0/23
Jilesen et al. [33]	8/51	0/21
Dokmak et al. [35]	25/102	10/63
Boggi et al. [30]		

CP: central pancreatectomy; DP: distal pancreatectomy.

## Data Availability

The data types used to support the findings of this study are included in the article and supplementary information files.

## Additional Points

Significant heterogeneity existed in three continuous variables (operation time, blood loss, and length of hospital stay), which indicates the difference is surgical skills between surgeons, studies conducted in different countries, different sample sizes, and so on. This study lacks data for postoperative gastroparesis and only a few studies recorded data on tumor recurrence. Moreover, only prospective observational and retrospective studies were identified during the literature search, so this study may not be as reliable as meta-analysis was performed on clinical trials and international multicenter studies.

## Supplementary Materials

Supplementary Materials Bias figure “/” funnel plot: funnel plot for publication bias “data mean”: collected data for meta-analysis. Supplementary Figure 1: forest plot comparing perioperative mortality for central versus distal pancreatectomy. Supplementary Figure 2: forest plot comparing postoperative insulin-dependent diabetes mellitus (IDDM) for central versus distal pancreatectomy. Supplementary Figure 3: forest plot comparing tumor recurrence for central versus distal pancreatectomy.
