# Supplementary figures and images for: Overall Postoperative Morbidity and Pancreatic Fistula Are Relatively Higher after Central Pancreatectomy than Distal Pancreatic Resection: A Systematic Review and Meta-Analysis
Source: Biomed Res Int. 2020 Feb 22;2020:7038907. doi: 10.1155/2020/7038907 (PMC7057026; doi:10.1155/2020/7038907)

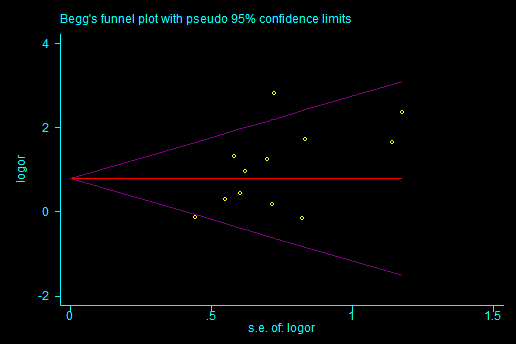

Supplement: Supplementary Materials — Bias figure “/” funnel plot: funnel plot for publication bias “data mean”: collected data for meta-analysis. Supplementary Figure 1: forest plot comparing perioperative mortality for central versus distal pancreatectomy. Supplementary Figure 2: forest plot comparing postoperative insulin-dependent diabetes mellitus (IDDM) for central versus distal pancreatectomy. Supplementary Figure 3: forest plot comparing tumor recurrence for central versus distal pancreatectomy. [file 7038907.f1.zip › 7038907.f1/bias figure/clinically relevant pancreatic fistula.tif]

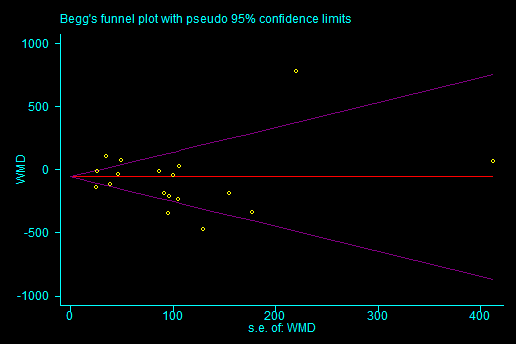

Supplement: Supplementary Materials — Bias figure “/” funnel plot: funnel plot for publication bias “data mean”: collected data for meta-analysis. Supplementary Figure 1: forest plot comparing perioperative mortality for central versus distal pancreatectomy. Supplementary Figure 2: forest plot comparing postoperative insulin-dependent diabetes mellitus (IDDM) for central versus distal pancreatectomy. Supplementary Figure 3: forest plot comparing tumor recurrence for central versus distal pancreatectomy. [file 7038907.f1.zip › 7038907.f1/bias figure/intraoperative blood loss.tif]

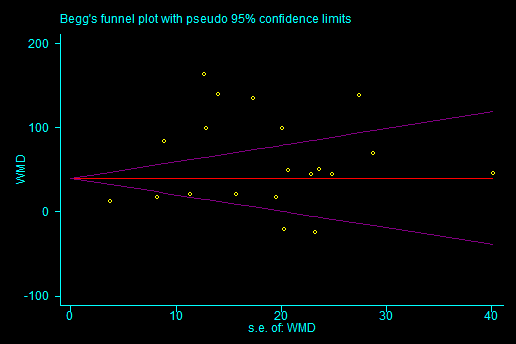

Supplement: Supplementary Materials — Bias figure “/” funnel plot: funnel plot for publication bias “data mean”: collected data for meta-analysis. Supplementary Figure 1: forest plot comparing perioperative mortality for central versus distal pancreatectomy. Supplementary Figure 2: forest plot comparing postoperative insulin-dependent diabetes mellitus (IDDM) for central versus distal pancreatectomy. Supplementary Figure 3: forest plot comparing tumor recurrence for central versus distal pancreatectomy. [file 7038907.f1.zip › 7038907.f1/bias figure/operation time.tif]

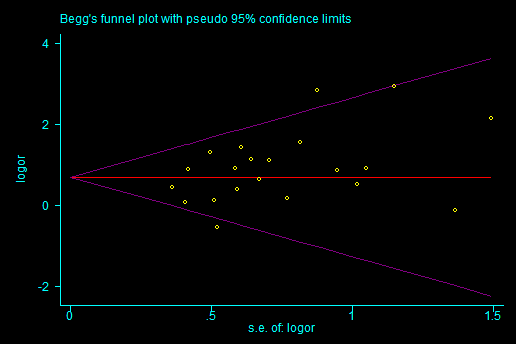

Supplement: Supplementary Materials — Bias figure “/” funnel plot: funnel plot for publication bias “data mean”: collected data for meta-analysis. Supplementary Figure 1: forest plot comparing perioperative mortality for central versus distal pancreatectomy. Supplementary Figure 2: forest plot comparing postoperative insulin-dependent diabetes mellitus (IDDM) for central versus distal pancreatectomy. Supplementary Figure 3: forest plot comparing tumor recurrence for central versus distal pancreatectomy. [file 7038907.f1.zip › 7038907.f1/bias figure/overall morbidity.tif]

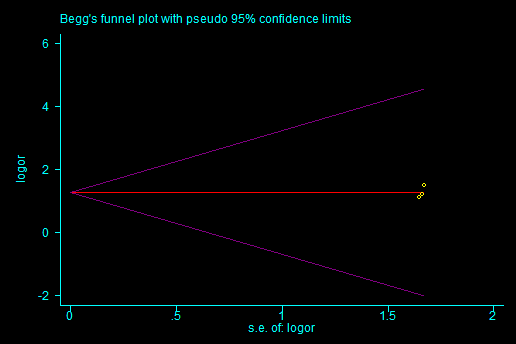

Supplement: Supplementary Materials — Bias figure “/” funnel plot: funnel plot for publication bias “data mean”: collected data for meta-analysis. Supplementary Figure 1: forest plot comparing perioperative mortality for central versus distal pancreatectomy. Supplementary Figure 2: forest plot comparing postoperative insulin-dependent diabetes mellitus (IDDM) for central versus distal pancreatectomy. Supplementary Figure 3: forest plot comparing tumor recurrence for central versus distal pancreatectomy. [file 7038907.f1.zip › 7038907.f1/bias figure/overall mortality.tif]

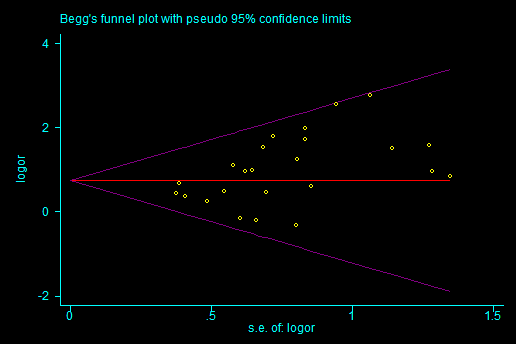

Supplement: Supplementary Materials — Bias figure “/” funnel plot: funnel plot for publication bias “data mean”: collected data for meta-analysis. Supplementary Figure 1: forest plot comparing perioperative mortality for central versus distal pancreatectomy. Supplementary Figure 2: forest plot comparing postoperative insulin-dependent diabetes mellitus (IDDM) for central versus distal pancreatectomy. Supplementary Figure 3: forest plot comparing tumor recurrence for central versus distal pancreatectomy. [file 7038907.f1.zip › 7038907.f1/bias figure/overall pancreatic fistula.tif]

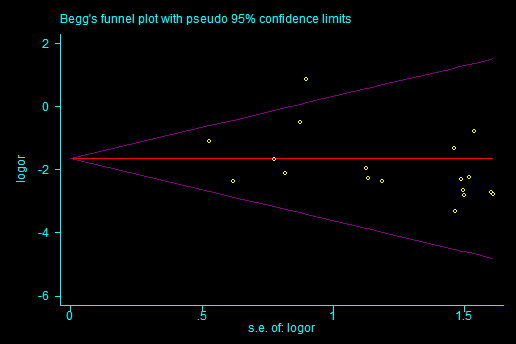

Supplement: Supplementary Materials — Bias figure “/” funnel plot: funnel plot for publication bias “data mean”: collected data for meta-analysis. Supplementary Figure 1: forest plot comparing perioperative mortality for central versus distal pancreatectomy. Supplementary Figure 2: forest plot comparing postoperative insulin-dependent diabetes mellitus (IDDM) for central versus distal pancreatectomy. Supplementary Figure 3: forest plot comparing tumor recurrence for central versus distal pancreatectomy. [file 7038907.f1.zip › 7038907.f1/bias figure/postoperative endocrine insufficiency.tif]

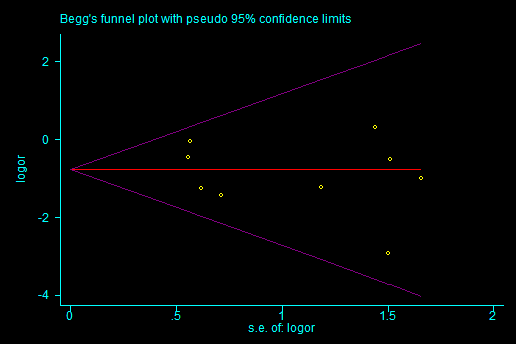

Supplement: Supplementary Materials — Bias figure “/” funnel plot: funnel plot for publication bias “data mean”: collected data for meta-analysis. Supplementary Figure 1: forest plot comparing perioperative mortality for central versus distal pancreatectomy. Supplementary Figure 2: forest plot comparing postoperative insulin-dependent diabetes mellitus (IDDM) for central versus distal pancreatectomy. Supplementary Figure 3: forest plot comparing tumor recurrence for central versus distal pancreatectomy. [file 7038907.f1.zip › 7038907.f1/bias figure/postoperative exocrine insufficiency.tif]

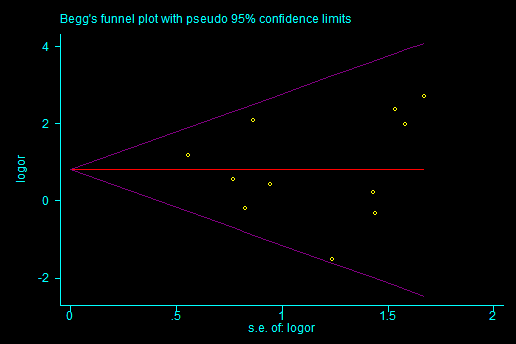

Supplement: Supplementary Materials — Bias figure “/” funnel plot: funnel plot for publication bias “data mean”: collected data for meta-analysis. Supplementary Figure 1: forest plot comparing perioperative mortality for central versus distal pancreatectomy. Supplementary Figure 2: forest plot comparing postoperative insulin-dependent diabetes mellitus (IDDM) for central versus distal pancreatectomy. Supplementary Figure 3: forest plot comparing tumor recurrence for central versus distal pancreatectomy. [file 7038907.f1.zip › 7038907.f1/bias figure/postoperative hemorrhage.tif]

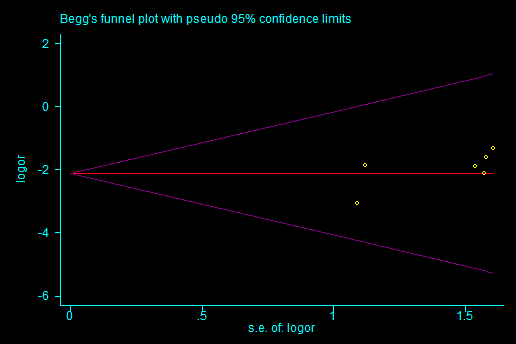

Supplement: Supplementary Materials — Bias figure “/” funnel plot: funnel plot for publication bias “data mean”: collected data for meta-analysis. Supplementary Figure 1: forest plot comparing perioperative mortality for central versus distal pancreatectomy. Supplementary Figure 2: forest plot comparing postoperative insulin-dependent diabetes mellitus (IDDM) for central versus distal pancreatectomy. Supplementary Figure 3: forest plot comparing tumor recurrence for central versus distal pancreatectomy. [file 7038907.f1.zip › 7038907.f1/bias figure/postoperative insulin dependant diabetes mellitus.tif]

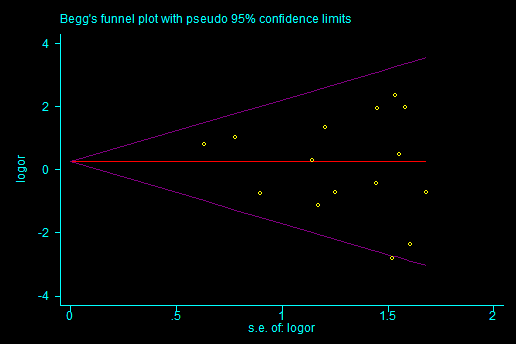

Supplement: Supplementary Materials — Bias figure “/” funnel plot: funnel plot for publication bias “data mean”: collected data for meta-analysis. Supplementary Figure 1: forest plot comparing perioperative mortality for central versus distal pancreatectomy. Supplementary Figure 2: forest plot comparing postoperative insulin-dependent diabetes mellitus (IDDM) for central versus distal pancreatectomy. Supplementary Figure 3: forest plot comparing tumor recurrence for central versus distal pancreatectomy. [file 7038907.f1.zip › 7038907.f1/bias figure/reoperation.tif]

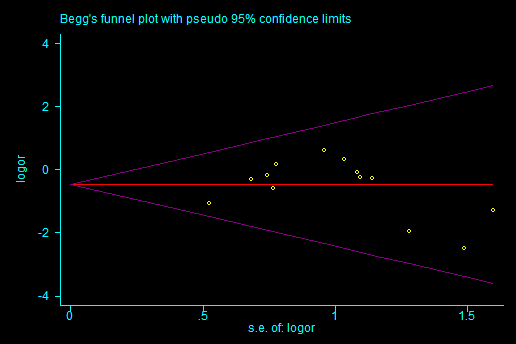

Supplement: Supplementary Materials — Bias figure “/” funnel plot: funnel plot for publication bias “data mean”: collected data for meta-analysis. Supplementary Figure 1: forest plot comparing perioperative mortality for central versus distal pancreatectomy. Supplementary Figure 2: forest plot comparing postoperative insulin-dependent diabetes mellitus (IDDM) for central versus distal pancreatectomy. Supplementary Figure 3: forest plot comparing tumor recurrence for central versus distal pancreatectomy. [file 7038907.f1.zip › 7038907.f1/bias figure/transfusion requirement.tif]

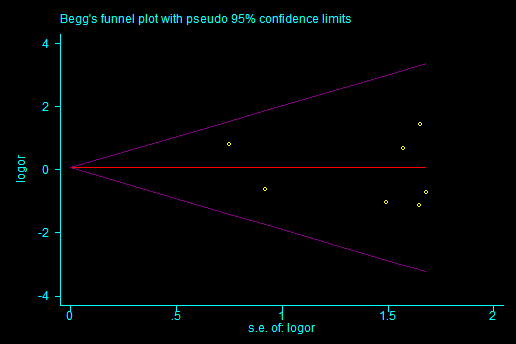

Supplement: Supplementary Materials — Bias figure “/” funnel plot: funnel plot for publication bias “data mean”: collected data for meta-analysis. Supplementary Figure 1: forest plot comparing perioperative mortality for central versus distal pancreatectomy. Supplementary Figure 2: forest plot comparing postoperative insulin-dependent diabetes mellitus (IDDM) for central versus distal pancreatectomy. Supplementary Figure 3: forest plot comparing tumor recurrence for central versus distal pancreatectomy. [file 7038907.f1.zip › 7038907.f1/bias figure/tumor recurrance.tif]
